# Different Spectral Analysis Methods for the Theta/Beta Ratio Calculate Different Ratios But Do Not Distinguish ADHD from Controls

**DOI:** 10.1007/s10484-020-09471-2

**Published:** 2020-05-20

**Authors:** Hanneke van Dijk, Roger deBeus, Cynthia Kerson, Michelle E. Roley-Roberts, Vincent J. Monastra, L. Eugene Arnold, Xueliang Pan, Martijn Arns

**Affiliations:** 1 Research Institute Brainclinics, Brainclinics Foundation, Bijleveldsingel 32, 6524 AD Nijmegen, The Netherlands; 2 Department of Psychology, University of North Carolina, Asheville, USA; 3 Saybrook University, Oakland, CA USA; 4 APEd, Napa, CA USA; 5 Department of Psychiatry, Creighton University, Omaha, NE USA; 6 FPI Attention Disorders Clinic, Endicott, NY USA; 7 Department of Biomedical Informatics, Ohio State University, Columbus, OH USA; 8 Department of Experimental Psychology, Utrecht University, Utrecht, The Netherlands; 9 neuroCare Group, Munich, Germany

**Keywords:** ADHD, ICAN, iSPOT-A, Theta-Beta-Ratio, Spectral analysis methods, EEG

## Abstract

There has been ongoing research on the ratio of theta to beta power (Theta/Beta Ratio, TBR) as an EEG-based test in the diagnosis of ADHD. Earlier studies reported significant TBR differences between patients with ADHD and controls. However, a recent meta-analysis revealed a marked decline of effect size for the difference in TBR between ADHD and controls for studies published in the past decade. Here, we test if differences in EEG processing explain the heterogeneity of findings. We analyzed EEG data from two multi-center clinical studies. Five different EEG signal processing algorithms were applied to calculate the TBR. Differences between resulting TBRs were subsequently assessed for clinical usability in the iSPOT-A dataset. Although there were significant differences in the resulting TBRs, none distinguished between children with and without ADHD, and no consistent associations with ADHD symptoms arose. Different methods for EEG signal processing result in significantly different TBRs. However, none of the methods significantly distinguished between ADHD and healthy controls in our sample. The secular effect size decline for the TBR is most likely explained by factors other than differences in EEG signal processing, e.g. fewer hours of sleep in participants and differences in inclusion criteria for healthy controls.

## Introduction

Attention deficit hyperactivity disorder (ADHD) is a disorder characterized by inattention, impulsivity and/or hyperactivity and is the most common of childhood psychiatric disorders. Many studies have investigated EEG in children with ADHD compared to those of children without ADHD. Ever since the first description of deviant fronto-central slow-wave EEG activity (i.e., *at frequencies of 5–6/s*) in ‘behavioral problem children’ (Jasper et al. 1938), which was included in the 4–8/s band later termed ‘theta activity’ (Walter and Dovey 1944), excess theta EEG power has often been reported in patients with ADHD (Arns et al. 2013). Others have proposed the ratio of theta to beta, or Theta/Beta Ratio (TBR), to be a better metric to differentiate between children with and without ADHD (Monastra et al. 1999, 2001). However, a recent meta-analysis could not confirm this measure to be a reliable diagnostic metric in ADHD as the effect sizes reported for the differences in TBR for ADHD and controls demonstrated significant heterogeneity (Arns et al. 2013). The authors found this heterogeneity to be explained by year of publication: the TBR increased for the non-ADHD groups over the last two decades, possibly as a result of reduced sleep duration (Arns et al. 2013; Bijlenga et al. 2019). Additionally, they suggested that differences in EEG equipment and software, including filtering techniques and EEG processing details (e.g. tapering or averaging) could explain the differences. That suggestion has not been tested in detail since that meta-analysis. Therefore, in the current paper we investigate if differences in signal processing could explain the null finding in most recent studies and could be the source of heterogeneity in the meta-analysis. For this purpose we analyzed screening data from the ICAN (International Collaborative ADHD Neurofeedback) multisite clinical trial preceding treatment, where a fixed rule of TBR ≥ 4.5 was used as an inclusion criterion (The Collaborative Neurofeedback Group 2013). We also investigated the clinical relevance of the TBR by applying the various permutations of computational methods to data from the iSPOT-A study (International Study to Predict Optimized Treatment in ADHD), the largest combined sample of QEEG recordings of children with ADHD (N = 336) and without ADHD (N = 158; Arns et al. 2018). We used these data specifically because a previous study found no significant differences in TBR between groups with and without ADHD (Arns et al. 2018). Finally, the TBR was correlated to age, which has been widely validated (Arns et al. 2013), and ADHD symptoms using the various metrics. Those metrics were compared to identify which specific processing technique is most biologically relevant.

## Methods

### Data and Recordings

Data for this study were acquired during screening of the ICAN study (The Collaborative Neurofeedback Group 2013) and the iSPOT-A study (Arns et al. 2018) prior to treatment.

#### ICAN Study

For the resting state (passively sitting with eyes open for 2 min) analysis, the QEEGs recorded from electrodes Fz and Cz were used, in line with the electrode sites most often used in TBR research (Arns et al. 2013). Only QEEGs from children (7–10 years old) with a TBR ≥ 4.5 (N = 272) were included in the analysis based on the norms by Monastra et al (1999) and Snyder and Hall (2006). Further details of the study rationale and protocol have been published elsewhere (The Collaborative Neurofeedback Group 2013).

#### iSPOT-A Study

The iSPOT-A dataset (Arns et al. 2018) consisted of 336 subjects with and 158 subjects without ADHD. The iSPOT-A study was a phase-IV, multi-site, international, open-label effectiveness trial in which ADHD patients were prescribed methylphenidate (MPH), from seven international research sites. Full details of the study protocol have been published elsewhere (Elliott et al. 2017). Further details of the study rationale and protocol have been published elsewhere (Arns et al. 2018). Selecting the subjects for whom the (2-min eyes open) EEG was available resulted in 328 ADHD patients and 151 control subjects (age: 11.98 ± 3.27 years; male: 72.4%). The EEG channels Fz and Cz were used for further analysis.

### Analysis

All analyses were performed using MATLAB (MathWorks Inc.) and the EEG/MEG analysis MATLAB-toolbox Fieldtrip (Oostenveld et al. 2011). EEG data were high pass filtered from 0.3 Hz, lowpass filtered at 60 Hz and cut into 2-s epochs of artifact-free segments, resulting in 45 epochs per subject. For ICAN data the automated de-artifacting from the “Monastra-Lubar ADHD Assessment Suite” and add-on to the Thought Technology (TT) software suite was used; for iSPOT-A data the automated de-artifacting detailed in Arns et al. (2016), was used. To compute the TBR one needs to compute the power in the theta as well as the beta band. Since methods for estimating the spectral power were not always clearly described we estimated the spectral power with methods that are most commonly used in the literature. Most spectral analysis variations presented in this study are therefore based on the Fast Fourier Transform (FFT; Cooley and Tukey 1965) since this was the most common method used by studies described in the meta-analysis by Arns et al. (2013). The FFT decomposes the EEG time series activation into sinusoidal functions with different amplitudes and frequencies. The FFT assumes that the data are recorded for an infinite amount of time and fits sinusoidal functions of integer amount of cycles and the two endpoints are interpreted as though they were connected. When the data are discontinuous, this will result in high-frequency components in the FFT power spectrum (spectral leakage), that are not in the original data. Recorded EEG data are never infinite, (e.g. when the EEG is divided into epochs), therefore a taper is normally applied: The two endpoints are tapered off to make them meet, avoiding the high-frequency artifacts. In the current study, among other issues, the effect of different tapering types on the TBR are explored.

#### Base Computation

The initial aim of the current study was to compare the different computational methods that were applied to obtain the TBR in the studies included in the meta-analysis (Arns et al. 2013); however, it was not possible to trace back a detailed description of said methods. Therefore, the base computation entailed the most commonly used methods (including several variations) for computing low frequency power spectral densities. Power spectral density estimations were computed using the FFT with a Hann taper (Blackman and Tukey 1958) of 2 s (0.5 Hz frequency resolution), to avoid the spectral leakage described above. The Hann taper thus covered the entire epoch containing ~ 10 cycles of theta range frequencies and ~ 36 cycles in the beta frequency range. The theta range was defined as frequencies between 4–8 Hz; the beta range was defined as between 13–21 Hz, in line with the approach advocated by Monastra et al. (1999). The average spectral power in the theta range was then divided by the average power in the beta range, resulting in the Theta/Beta Ratio (TBR).

#### Computational Variations

A short overview of the following computational variations is depicted in Table 1.

**Table 1:** The five different methods that were investigated are described here, with bold indicating how the method is referred through in the text

Method	Preprocessing	Frequency analysis	Output	Average
1	Uncorrected	FFT (Hann)	uV^2^	**Trial**
2	Uncorrected	FFT (Hann)	uV^2^	Session
3	**1/f corrected**	FFT (Hann)	uV^2^	Session
4	Uncorrected	**Multitaper**	uV^2^	Session
5	Uncorrected	**Welch (Hann)**	uV^2^	Session

**Fig. 1:**
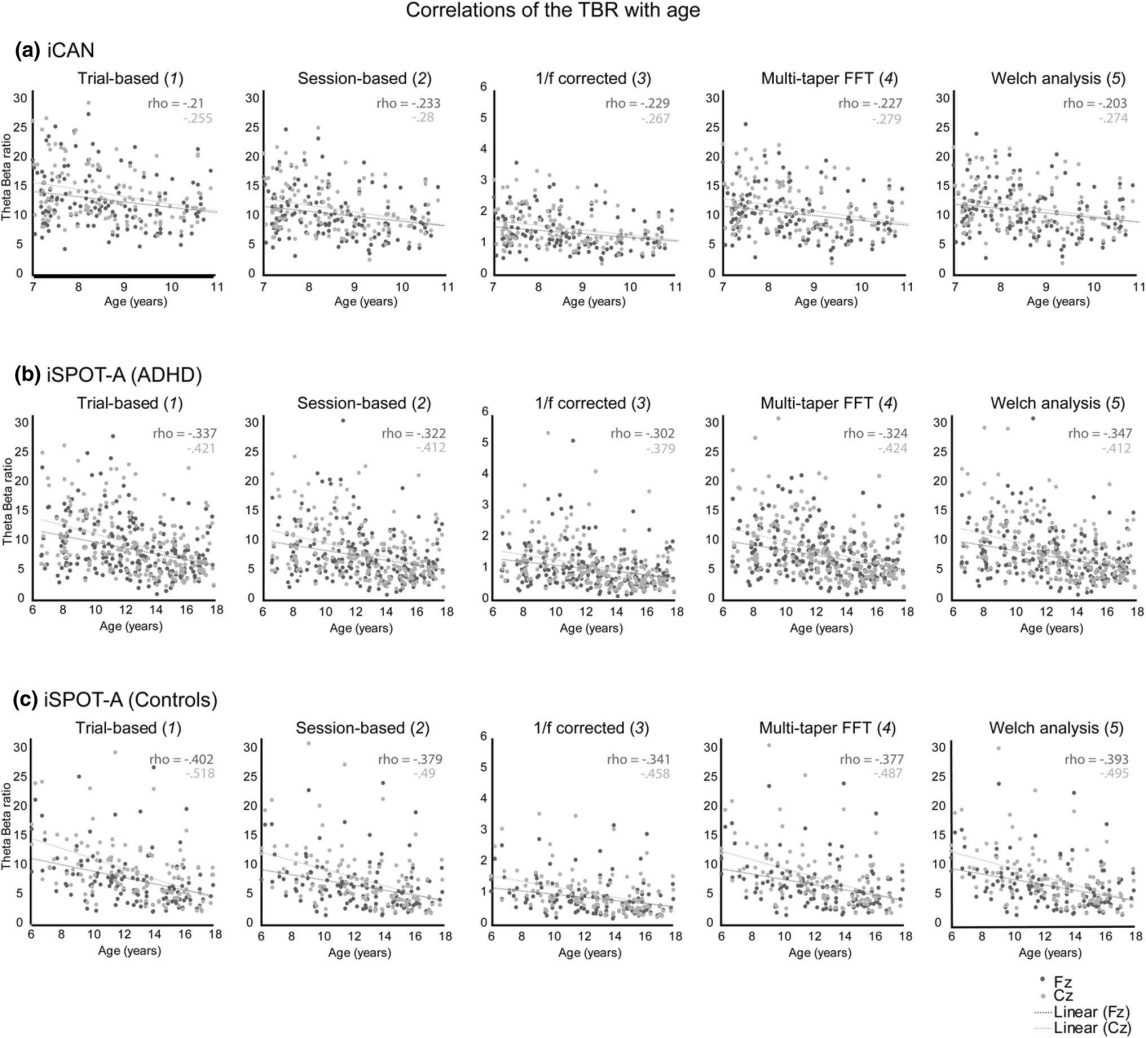
Correlations between the TBR and age for all methods described in Table 1 for both the ICAN and iSPOT-A datasets. All Spearman correlations were rho > − 0.2 (p < .001; DF = 234) for the ICAN dataset. In the control subjects as well as in the ADHD patients in the iSPOT-A dataset rho > − 0.4 (p < .001; DF = 123 and DF = 219 respectively)

The numbers in the first column, representing the different methods, are referred to in the following tables and Fig. 1

#### Preprocessing Variations

##### Uncorrected

In this variation we did not do any further preprocessing or corrections.

##### Removal of Pink Noise (1/f Noise)

The power per frequency interval is inversely proportional to the frequency of the signal. Pink- or 1/f noise, is very common in biological signals (Szendro et al. 2001) and is more dominant at lower frequencies than at higher frequencies. In experimental research, when comparing the spectral power between two conditions, we normally do not correct explicitly for pink noise, as this noise disappears in the contrast. However, there are cases where it is valid to correct for it, such as detecting peaks that sit on a 1/f flank. Since the frequency range of theta (4–8 Hz) is considerably lower than that of beta (13–21 Hz) this means that a larger part of the spectral energy in the theta band can be explained by pink noise than in the beta band. Therefore, we also investigated the TBR resulting from the power spectral estimation after correcting for the pink (1/f) noise (see Eq. 1) in the input for the FFT.

1 $$f\left(t\right)=\mathrm{sin}\left(w*t\right)$$

$$\frac{df}{dt}=w*\mathrm{cos}(w*t)$$

Taking the derivative of a sine multiplies the output with *w*, which is the frequency (ƒ) in radians per second (t). This is a linear operation and since the FFT assumes that the data is the sum of many sine waves, taking the derivative of the data is equivalent to taking the derivative of all separate sine-wave contributions to the data. The consequence is that the derivative in time results in the Fourier spectrum being multiplied by f (for any frequency). As a consequence, the 1/f effect in the spectrum is counteracted by a 1*f effect of the time-domain derivative, resulting in the power-spectrum being normalized for the 1/f noise, much like the subtraction of a regression model of the 1/f noise.

#### Spectral analysis Methods

##### Welch’s Method

The Welch method is based on the concept of using power spectral density (PSD) estimates, which are the result of converting a signal from the time domain to the frequency domain. Welch's method is an improvement on the standard spectral density estimating method, in that it reduces noise in the estimated power spectra. Due to the noise caused by imperfect and finite data, the noise reduction from Welch's method is often desired (Welch 1967). The method divides the time series into (possibly overlapping) segments, computing a modified spectrogram for each segment, and then averaging the PSD estimates. This method decreases the variance of the estimate compared to a single spectrogram estimate of the entire data record. However, note that this method results in reduced resolution of the estimator, meaning there is a tradeoff between the reduction in variance and frequency resolution. For the Welch’s method analysis in this study we used 50% overlapping Hann tapers of 0.5 s to estimate the PSD in both theta and beta frequency ranges.

##### Multi-taper Method

Another widely used method for spectral density estimation is the multi-taper method (Thomson 1982). This method is typically used in order to achieve better control over the frequency resolution. The FFT spectral density estimation is assumed to be a reliable representation of the amplitude and relative phase of the corresponding component frequency. This cannot always be assumed, for instance when single trial estimates are only noisy reflections of the underlying process, and the PSD obtained with the Fourier transform is a biased estimate of the true spectral content, depending among others on the chosen time-window length, or the tapering function. Normally, this is corrected by averaging over many instances of the event. Alternatively, the multi-taper method uses multiple orthogonal tapers [or discrete prolate spheroidal sequences (DPSS; Thomson 1982)] to obtain many independent estimates from the same sample. Finally, for each sample all of the independently tapered estimates are averaged.

##### Wavelet Analysis

Contrary to the above described methods, having a fixed taper length, the time windows that are used for wavelet analysis are dependent on the frequency. A wavelet decomposition uses a wave-like scalable function that is well localized in both time and frequency. Spectral density estimations for lower frequencies are computed using larger (time) windows than for higher frequencies. This approach calculates power spectral densities with time windows that depend on frequency, using a taper with Gaussian shape. Thus, it does not convolve the data with sine waves like the FFT, but with Mortlet (or Gabor) wavelets (Meyer 1993). Using this method, we computed the power spectral density with a wavelet length of 7 cycles. For example, for 5Hz the time-window length is: 200 ms*7 cycles = 1.4 s and for 15Hz the time-window would be 467 ms. Resulting in a PSD estimation of all frequencies computed from a similar relative amount of input data, and; therefore, relatively the same frequency resolution (Le Van Quyen and Bragin 2007).

##### Session Average

Average theta power and beta power over the complete session and then compute the ratio between the two (see Eq. 2).

2 $${TBR}_{session}=\frac{\stackrel{-}{f\tau }}{\stackrel{-}{f\beta }}$$

##### Trial Average

This method could be used to normalize for temporal fluctuations in the EEG. By computing the ratio on the trial level, temporal differences in background activity are less likely to influence the resulting TBR, than averaging over the complete session. We perform this analysis by computing the ratio between theta and beta power for each 2-s epoch and then compute the average ratio over the complete session (see Eq. 3). This allows for correcting possible unequal fluctuations in theta and beta power over time.

3 $${TBR}_{trial}= \frac{\sum_{i=0}^{n}\frac{{f\tau }_{trial}}{{f\beta }_{trial}}}{n}$$

where *n *is the number of trials.

After computing the TBR for all different variations for all subjects individually we compared the output of the different methods using Cohen’s d as an effect size measure.

### Clinical Implications; Testing the TBR in ADHD

In addition to computing the various TBR computations, we investigated if any of the variations were of diagnostic value in ADHD. For this we used the iSPOT-A dataset consisting of EEGs of 328 subjects with and 151 subjects without ADHD. We selected 5 methods for computing the TBR that showed a difference with the outcome of the other methods in the ICAN sample and computed these five types of TBR for each individual. We tested for statistical differences in the TBR between the ADHD and controls in the iSPOT-A cohort using unpaired t-tests and for clinical differences using Cohen’s *d*.

As a final step, the different TBR measures were correlated with age (Spearmans’ Rho) to test for possible differences in this well-known and widely acknowledged association (Bresnahan et al. 1999; Liechti et al. 2013). Additionally, we correlated the TBR with inattention and hyperactivity-impulsivity in the ICAN sample (covaried for age) to inspect dimensional associations. To investigate robustness, the correlations between the TBR measures and age, inattention and hyperactivity (parent- and teacher ratings; CPRS:L, Conners et al. 1998) were replicated in the iSPOT-A sample.

## Results

In this study we investigated various methods (depicted in Table 1) to compute the TBR and compared the outputs. The results are depicted in Table 2. When comparing the TBRs resulting from the different FFT permutations and numerically comparing the methods, we observed a medium sized difference in TBR between the trial ratio based TBR ($${TBR}_{trial})$$ and session average ratio TBR ($${TBR}_{session}$$; for Cz: *d* = 0.407 ± 0.08, *r*^*2*^ = 0.931). The TBR resulting from the 1/f-noise-removed analysis showed a large difference from the TBR resulting from the uncorrected analysis (i.e. for Cz: *d* = 2.338 ± 0.11, *r*^*2*^ = 0.897). Comparing the different spectral analysis methods, we observed an effect size of *d* = − 0.011 ± 0.09 at Fz (*r*^*2*^ = 0.902) between the FFT and multi-taper analysis, and *d* = − 0.097 ± 0.09 at Fz (*r*^*2*^ = 0.872) between the FFT and Welch analysis.

**Table 2:** Comparisons between the TBRs computed using the five different methods described in Table 1

Comparison of the different TBR computation methods				
	Fz		Cz	
	d (SE)	R^2^	d (SE)	R^2^
Session vs Trial (*1 vs 2)*	0.407 (0.08)	0.916	0.402 (0.08)	0.931
Uncorrected vs 1/f (*2 vs 3)*	2.384 (0.11)	0.885	2.338 (0.11)	0.897
FFT(Hann) vs Multitaper *(2 vs 4)*	− 0.011 (0.09)	0.902	0.001 (0.09)	0.919
FFT(Hann) vs Welch (Hann) *(2 vs 5)*	− 0.097 (0.09)	0.872	− 0.032 (0.09)	0.884

The numbers in the first column refer to methods described in Table 1

### Implications for Clinical Practice

The five methods we described in the previous analysis were used to test whether the resulting TBRs showed differences between ADHD and non-ADHD groups within the iSPOT-A dataset. The selected methods are depicted in Table 1. As shown in Table 3 and Fig. 1 there is no clinically useful difference in any of the TBR computations between the ADHD and non-ADHD groups. The ES of the difference in TBR between ADHD and non-ADHD ranged between *d*(Cz) = 0.102 for method 5 and maximal *d*(Fz) = 0.215 for method 3. None of the group differences reached significance (all *p* > 0.062, without correction for multiple tests) .

**Table 3:** The results for the comparisons between the TBR for ADHD and Controls (iSPOT), for the five methods presented in Table 1

Method	Mean TBR (SD)							
	Fz				Cz			
	ADHD	Controls	*d*	*p*	ADHD	Controls	*d*	*p*
1	8.77 (4.7)	7.88 (4.6)	0.191	.092	9.75 (5.3)	9.20 (5.5)	0.104	.356
2	7.46 (5.0)	6.73 (4.2)	0.204	.073	8.42 (4.8)	7.90 (4.9)	0.106	.347
3	1.01 (0.6)	0.88 (0.6)	0.215	.062	1.12 (0.7)	1.03 (0.6)	0.134	.244
4	7.66 (4.4)	7.80 (4.1)	0.200	.079	8.54 (4.2)	7.97 (4.8)	0.117	.299
5	7.65 (4.2)	6.89 (3.7)	0.187	.101	8.62 (4.7)	8.14 (4.6)	0.102	.366

Mean TBRs are presented with the standard deviations (SD). Cohen’s d was computed for the differences between ADHD and Controls, and the means were statistically tested for difference with a two-sample t-test

In the ICAN data, Spearman correlational analyses between age and TBR for all permutations showed highly significant correlations (all Cz *p* < 0.005 unless stated otherwise), as expected, with the strongest correlations for the Session-based averaging method (*r* = − 0.28; *df* = 234) followed by Welch (Hann) (*r* = − 0.27; *df* = 234) and the weakest correlation for Trial based (*r* = − 0.26; *df* = 234). Effectively, this implicates a 1.3% difference in explained variance (6.4–5.5%) between the best and worst permutation for the known association between TBR and age.

No significant partial correlations (covaried for age) were found for parent-rated inattention or hyperactivity/impulsivity (all *p* > 0.1) ratings or for teacher-rated inattention. For teacher rated hyperactivity/impulsivity a significant correlation was found for Welch (Hann) TBR (Fz: *p* = 0.04; *rho* = − 0.179; *df* = 165), which does not survive Bonferroni correction. Note the significant teacher-rated correlation is the opposite to what was expected, in that high TBR is associated with *low* levels of inattention and hyperactivity/impulsivity. With a sample that was adequately powered, the lack of significant correlation casts doubts on the association of TBR with severity of ADHD symptoms. Therefore, we performed the same correlations within the larger iSPOT-A samples as well.

For iSPOT-A significant correlations (all for site Cz unless stated otherwise) with age (all *p* < 0.001) were found as expected, with the strongest correlations for Trial based (controls: *r* = − 0.518; *df* = 123; ADHD: *r* = − 0.421; *df* = 219); Multi-taper (controls: *r* = − 0.487; *df* = 123; ADHD: *r* = − 0.424; *df* = 219) and Welch (controls: *r* = − 0.495; *df* = 123; ADHD: *r* = − 0.432; *df* = 219).

No correlations were found between any of the TBR calculation methods and inattention or Hyperactivity/Impulsivity within the non-ADHD group (all *p* > 0.3). For the ADHD group, the most significant correlations (Cz unless stated otherwise) were for Inattention and Trial based averaging (@Fz, *p* = 0.022; *r* = 0.154; *df* = 216) and the lowest correlation for Welch (Hann) (*p* = 0.093; *r* = 0.114; *df* = 216). The explained variance ranged from 1.5 to 0.7%, effectively making a 0.8% difference of the explained variance in the association between TBR and inattention. For hyperactivity in the ADHD group, similar patterns were observed with the highest correlation for Trial based averaging (@Fz, *p* = 0.033; *r* = 0.144; *df* = 216) and the lowest correlation for Welch (*p* = 0.01; *r* = 0.109; *df* = 216), effectively explaining 1.2% and 0.3% of the variance and thus a 0.9% difference of the variance, however, none of these correlations would have survived a correction for multiple testing e.g. Bonferroni correction.

## Discussion

This study further investigated the finding of heterogeneity in the results of EEG studies that compared the TBR in ADHD and non-ADHD populations, as earlier described by Arns et al. (2013). To this end, various EEG processing methods were applied to test, (1) the differences in EEG processing on absolute TBR values using data from the ICAN study, (2) differences in EEG processing and associations with age and behavior (inattention and hyperactivity/impulsivity ratings) in the ICAN study, and (3) effect of differences in EEG processing on differences in TBR, age, and ADHD symptoms between ADHD and non-ADHD groups in the iSPOT-A study.

### Effect of Different Preprocessing- and Spectral Analysis Methods on the TBR Value

Given the lack of detail on the actual computation of the TBR provided by the papers in the meta-analysis (Arns et al. 2013), we compared various processing methods that are generally used and representative of EEG research from both resting state studies (session-averaging), event-related oscillation research (trial-averaging), and other methods often applied in EEG studies for power spectral density estimation. In most traditional EEG studies theta and beta power are analyzed and averaged across epochs, and TBR is calculated by subsequently calculating the ratio between the two averages (here referred to as ‘session-average’). However, in studies focused on event-related oscillation calculations (such as ratios), averages are first calculated for each individual trial and subsequently averaged over trials (here referred to as ‘trial-average’). Interestingly, the absolute values between these two methods also varied considerably, with medium effect sizes; the trial-average method resulted in numerically higher TBR values (Fz: *d* = 0.41). The largest difference in absolute TBR values compared to the session average FFT was found for the 1/f corrected data, which is logical because due to the removal of the 1/f (pink) noise the ratio of theta-to-beta power approached 1 (as can be seen in Table 3; Fz: *d* = 2.38). In contrast, the different frequency analysis approaches such as FFT vs. Welch or tapering methods Hann vs. DPSS had no major impact on absolute values of TBR, with very small ES (Cz: *d* = 0.01).

### TBR as Discriminator Between ADHD and Non-ADHD Group

**Fig. 2:**
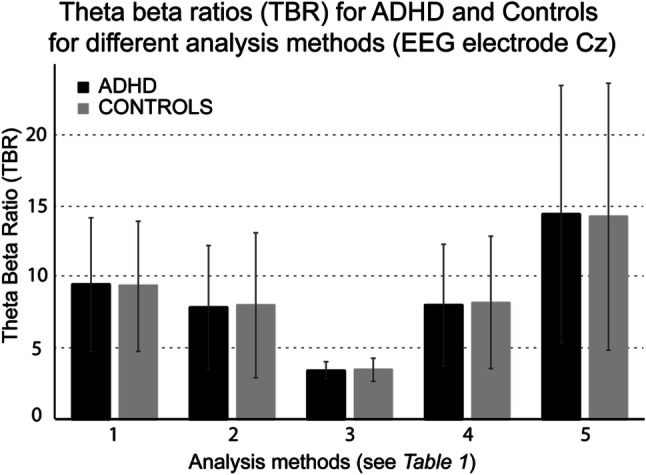
Theta-Beta ratio (TBR) for ADHD and Controls (iSPOT) for the different computational methods. The different TBRs are depicted for ADHD (black) and Controls (striped) for the computational methods described in Table 2. Error bars represent the standard error of the mean (SEM). Note the wide variation between computational systems contrasted with negligible differences between ADHD and normal controls within each method

Despite testing all of these commonly used frequency analyses there was no evidence that any of the resulting (numerically different) TBRs could distinguish between the ADHD and the non-ADHD group as visualized in Fig. 2. Correlational analyses showed the hypothesized strong correlation of all TBR metrics with age across both ICAN and iSPOT-A samples, supporting reliability and validity of the results. Overall, effects were rather comparable for all methods and contrasting the two most extreme methods (i.e. ‘strongest’ vs. ‘weakest’ correlation) made a difference of 2.5% of explained variance, showing the association between TBR and age is not impacted in a major way by the processing method. However, none of the described permutations demonstrated a group difference between ADHD and non-ADHD controls (see Fig. 1 and Table 3), with the largest between-group effect size being *d* = 0.204, normally considered a small effect size (Fz, for *Method 2*). Inconsistent associations with sum of the teacher-rated inattention and hyperactivity-impulsivity were found that (1) would not have survived correction for multiple testing (most significant correlation was p = 0.017); (2) were in the opposite-of-hypothesized direction in the ICAN sample (high TBR associated with low inattention and hyperactivity/impulsivity) and (3) were in the expected direction in the iSPOT-A sample, but with very low explained variance (max 2.6%), with no such association in the non-ADHD sample. In sum, TBR does not show a consistent association with ADHD symptoms. Our findings paired with the meta-analysis results suggest that TBR is not a useful stand-alone diagnostic tool for ADHD. Further, absolute TBR values might not be comparable from one software system to another (Kerson et al. 2020).

In addition to the lack of detail in the description of the process for computation of the TBR across studies, the specification of inclusion criteria for the “healthy” control groups in studies varies as well. In several of the earlier studies (Monastra et al. 1999, 2001) stringent criteria for normality were applied: non-clinical controls were required not to have met DSM-IV criteria for any psychiatric disorder, could not exhibit an atypical frequency of core ADHD symptoms on norm-referenced behavioral rating scales, and could not demonstrate an abnormal performance on a continuous performance test. The results of a QEEG examination were not included in the database unless the child had achieved at least 9 h of sleep and their physician attested in writing that the child did not have any medical condition associated with chronic impairment of attention (see listing of conditions in Monastra 2008). In the more recently conducted studies, the lack of control for medical conditions and sleep deficits in the sample has. led to the hypothesis that decreased sleep duration may be a primary factor accounting for the increased TBR in the control group over time (Arns et al. 2013; Bijlenga et al. 2019).

### Limitations

Limitations include incomplete investigation of all possible specific methods of calculating TBR. In particular, the specific combination of methods used by NEBA and the Thought Technology Monastra-Lubar ADHD suite could not be specifically assessed, and the EEGer method was not included. Rather than comparing different systems of hardware and software, this investigation used common underlying variants of algorithms utilized in different systems, representing a broad array of combinations. Another limitation is the correlational approach, which requires awareness of the sample to which the analysis is applied. For example, the ICAN sample had been selected for being 1.5 sd above the normal mean on the Conners three inattention scale and therefore represented only an extreme of one of the variables being correlated. It is possible that if the full range of both variables were available in the sample, a significant correlation might have been found between TBR and inattention. However, the inverse-of-expected trend for teacher ratings argues against that possibility.

## Conclusions

### The Use of TBR to Diagnose ADHD is Not Recommended

Looking at the patterns of results, no single measure arose as showing the most consistent ‘biologically relevant’ measure (i.e. one permutation that explained 5% more variance than others) that could inform future studies. For the age correlations, the Trial Based TBR and Welch method showed the strongest associations in both samples (7% in ICAN and 16–23% in iSPOT-A), with an average difference of 0.18%. So, although differences were overall small, a recommendation for future studies is to use the Trial-Based or Welch method for TBR studies.

*In summary*, with regard to the different EEG processing methods, we conclude that the significant heterogeneity observed in the meta-analysis by Arns et al. (2013) of recent papers is unlikely to be explained by differences in EEG processing methods.

## References

[CR100] Arns M, Bruder G, Hegerl U, Spooner C, Palmer D, Etkin A, Fallahpour K, Gatt J, Hirshberg L, Gordon E (2016). EEG alpha asymmetry as a gender-specific predictor of outcome to acute treatment with different antidepressant medications in the randomized iSPOT-D study. Clinical Neurophysiology.

[CR1] Arns M, Conners CK, Kraemer HC (2013). A decade of EEG Theta/Beta Ratio research in ADHD: A meta-analysis. Journal of Attention Disorders.

[CR2] Arns M, Vollebregt MA, Palmer D, Spooner C, Gordon E, Kohn M, Clarke S, Elliott GR, Buitelaar JK (2018). Electroencephalographic biomarkers as predictors of methylphenidate response in attention-deficit/hyperactivity disorder. European Neuropsychopharmacology.

[CR3] Bijlenga D, Vollebregt MA, Kooij JJS, Arns M (2019). The role of the circadian system in the etiology and pathophysiology of ADHD: time to redefine ADHD?. ADHD Attention Deficit and Hyperactivity Disorders.

[CR4] Blackman RB, Tukey JW (1958). The measurement of power spectra from the point of view of communication engineering-Part I. Bell System Technical Journal.

[CR5] Bresnahan, S. M., Anderson, J. W., & Barry, R. J. (1999). Age-related changes in quantitative EEG in attention-deficit/hyperactivity disorder. Biological Psychiatry, *46*, 1690–1697. https://s3.amazonaws.com/academia.edu.documents/46610380/s0006-3223_2899_2900042-620160619-2095-20jrqf.pdf?AWSAccessKeyId=AKIAIWOWYYGZ2Y53UL3A&Expires=1552304141&Signature=XkycW0qQ4lHh3dImabYpDEMAXVc%3D&response-content-disposition=inline%3Bfilename%3DAg 10.1016/s0006-3223(99)00042-6 10624551

[CR7] Conners CK, Sitarenios G, Parker JD, Epstein JN (1998). The Revised Conners’ Parent Rating Scale (CPRS-R): Factor structure, reliability, and criterion validity. Journal of Abnormal Child Psychology.

[CR8] Cooley JW, Tukey JW (1965). An Algorithm for the machine calculation of complex Fourier series. Mathematics of Computation.

[CR9] Elliott GR, Blasey C, Rekshan W, Rush AJ, Palmer DM, Clarke S, Kohn M, Kaplan C, Gordon E (2017). Cognitive testing to identify children with ADHD who do and do not respond to methylphenidate. Journal of Attention Disorders.

[CR10] Jasper HH, Solomon P, Bradley C (1938). Electroencephalographic analyses of behavior problem children. American Journal of Psychiatry.

[CR11] Kerson C, deBeus R, Lightstone H, Arnold LE, Barterian J, Pan X, Monastra VJ (2020). EEG Theta/Beta Ratio calculations differ between various EEG neurofeedback and assessment software packages: Clinical interpretation. Clinical EEG and Neuroscience.

[CR12] Le Van Quyen M, Bragin A (2007). Analysis of dynamic brain oscillations: Methodological advances. Trends in Neurosciences.

[CR13] Liechti MD, Valko L, Müller UC, Döhnert M, Drechsler R, Steinhausen H-C, Brandeis D (2013). Diagnostic value of resting electroencephalogram in attention-deficit/hyperactivity disorder across the lifespan. Brain Topography.

[CR14] Meyer, Y. (1993). Wavelets-algorithms and applications. In *Wavelets-Algorithms and applications*. Society for Industrial and Applied Mathematics. https://adsabs.harvard.edu/abs/1993ApMatM.

[CR15] Monastra, V. J. (2008). *Unlocking the potential of patients with ADHD : A model for clinical practice*. American Psychological Association. https://www.apa.org/pubs/books/4317139.aspx.

[CR16] Monastra VJ, Linden M, Lubar JF, Vandeusen P, Linden M, Green G, Philips A, Vandeusen P, Wing W, Fenger TN, Lubar JF, Vandeusen P (1999). Assessing attention deficit hyperactivity disorder via quantitative electroencephalography: An initial validation study. Neuropsychology.

[CR17] Monastra VJ, Lubar JF, Linden M, York N (2001). The development of a quantitative electroencephalographic scanning process for attention deficit-hyperactivity disorder: Reliability and validity studies. Neuropsychology.

[CR19] Oostenveld R, Fries P, Maris E, Schoffelen J-M (2011). FieldTrip: Open source software for advanced analysis of MEG, EEG, and invasive electrophysiological data. Computational Intelligence and Neuroscience.

[CR101] Snyder S, Hall J (2006). A meta-analysis of quantitative EEG power associated with attention-deficit hyperactivity disorder. Journal of Clinical Neurophysiology.

[CR20] Szendro P, Vincze G, Szasz A (2001). Pink-noise behaviour of biosystems. European Biophysics Journal.

[CR21] The Collaborative Neurofeedback Group (2013). A proposed multisite double-blind randomized clinical trial of neurofeedback for ADHD: Need, rationale, and strategy. Journal of Attention Disorders.

[CR22] Thomson DJ (1982). Spectrum estimation and harmonic analysis. Proceedings of the IEEE.

[CR23] Walter WG, Dovey VJ (1944). Electro-encephalography in cases of sub-cortical tumour. Journal of Neurology, Neurosurgery, and Psychiatry.

[CR24] Welch P (1967). The use of fast Fourier transform for the estimation of power spectra: A method based on time averaging over short, modified periodograms. IEEE Transactions on Audio and Electroacoustics.

